# Overview of Diverse Methyl/Alkyl-Coenzyme M Reductases and Considerations for Their Potential Heterologous Expression

**DOI:** 10.3389/fmicb.2022.867342

**Published:** 2022-04-25

**Authors:** Aleksei Gendron, Kylie D. Allen

**Affiliations:** Department of Biochemistry, Virginia Polytechnic Institute and State University, Blacksburg, VA, United States

**Keywords:** methyl-coenzyme M reductase, MCR, methanogens, anaerobic methanotrophic archaea, ANME

## Abstract

Methyl-coenzyme M reductase (MCR) is an archaeal enzyme that catalyzes the final step of methanogenesis and the first step in the anaerobic oxidation of methane, the energy metabolisms of methanogens and anaerobic methanotrophs (ANME), respectively. Variants of MCR, known as alkyl-coenzyme M reductases, are involved in the anaerobic oxidation of short-chain alkanes including ethane, propane, and butane as well as the catabolism of long-chain alkanes from oil reservoirs. MCR is a dimer of heterotrimers (encoded by *mcrABG*) and requires the nickel-containing tetrapyrrole prosthetic group known as coenzyme F_430_. MCR houses a series of unusual post-translational modifications within its active site whose identities vary depending on the organism and whose functions remain unclear. Methanogenic MCRs are encoded in a highly conserved *mcrBDCGA* gene cluster, which encodes two accessory proteins, McrD and McrC, that are believed to be involved in the assembly and activation of MCR, respectively. The requirement of a unique and complex coenzyme, various unusual post-translational modifications, and many remaining questions surrounding assembly and activation of MCR largely limit *in vitro* experiments to native enzymes with recombinant methods only recently appearing. Production of MCRs in a heterologous host is an important step toward developing optimized biocatalytic systems for methane production as well as for bioconversion of methane and other alkanes into value-added compounds. This review will first summarize MCR catalysis and structure, followed by a discussion of advances and challenges related to the production of diverse MCRs in a heterologous host.

## Introduction

**Figure 1:**
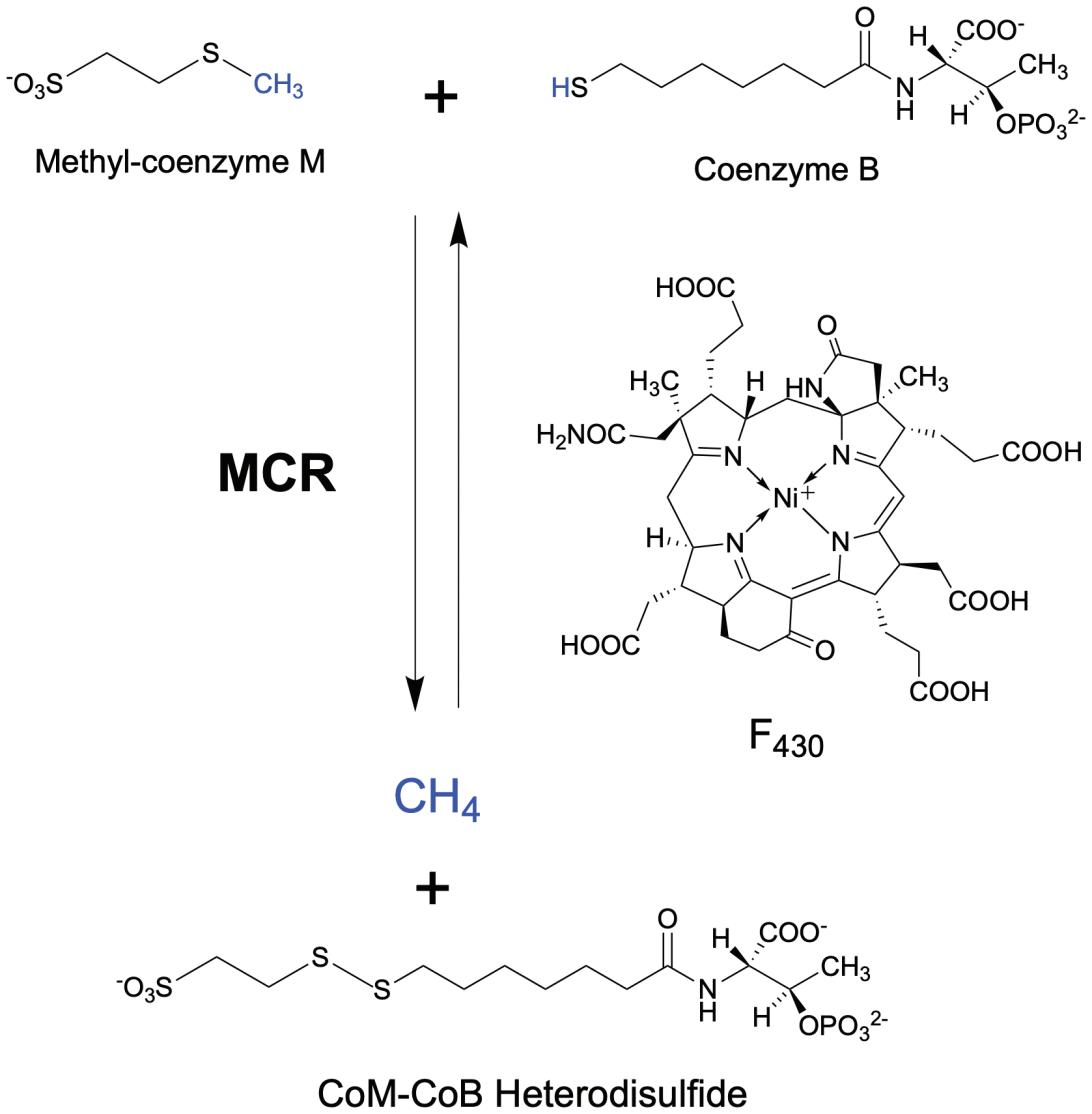
MCR-catalyzed reactions in the final step of methanogenesis in methanogens and the first step of the anaerobic oxidation of methane in anaerobic methanotrophs.

Methyl-coenzyme M reductase (MCR) catalyzes the final methane-forming step of methanogenesis in methanogens, and the initial methane activation step in the anaerobic oxidation of methane (AOM) in anaerobic methanotrophic archaea (ANME). MCR is generally highly conserved in sequence and structure among methanogens and ANME, where it consists of three different subunits, α (McrA), β (McrB), and γ (McrG), arranged in a *α*_2_*β*_2_*γ*_2_ configuration harboring two active sites (Ermler et al., 1997; Shima et al., 2012; Wagner et al., 2017). Each active site contains F_430_, the nickel hydrocorphin prosthetic group (Figure 1). Methanogenic MCR has been extensively studied in the methane formation direction, where it catalyzes the conversion of methyl-coenzyme M (CH_3_-S-CoM) and coenzyme B (HS-CoB) to methane and a CoM-S-S-CoB heterodisulfide (Bobik et al., 1987; Ellermann et al., 1987, 1988; Figure 1). This reaction is proposed to occur in reverse in ANME that anaerobically oxidize methane to CO_2_ *via* reverse methanogenesis (Hallam et al., 2004; Shima and Thauer, 2005; Scheller et al., 2010; Timmers et al., 2017). In addition to methane formation and oxidation, variants of MCR are also involved in the anaerobic oxidation of short- and long-chain alkanes (Borrel et al., 2019; Wang et al., 2021; Zhou et al., 2022).

Given the remarkable chemistry catalyzed by MCR as well as its central importance in the global carbon cycle and potential for bioenergy applications, this enzyme has been of interest to enzymologists since its initial discovery in the 1970s (McBride and Wolfe, 1971; Gunsalus and Wolfe, 1976). Much of what is known about MCR catalysis comes from work by several groups on the natively purified MCR from the methanogen, *Methanothermobacter marburgensis*. This organism grows to high cell densities (3 g dry mass per L) with a doubling time of less than 2 h (Kaster et al., 2011) and, most importantly, effective procedures have been developed for the isolation of an active MCR from this organism.

A major challenge in the MCR field is the heterologous production of recombinant MCRs. This is due to many reasons including, but not limited to, the heterooligomeric structure of MCR that may require chaperones for proper assembly, the requirement of the unique and complex coenzyme F_430_, the presence of several unusual post-translational modifications that are organism-specific, and the lack of knowledge surrounding proteins required for activation and incorporation of F_430_. Although undoubtedly a difficult task, successful development of heterologous expression systems for MCRs would transform the field, allowing further investigation into the catalytic properties and mechanistic aspects of different MCRs, as well as facilitate the development of optimized biocatalytic systems for methane production or methane conversion applications.

In this review, we will provide an overview of the most relevant aspects of MCR structure and catalysis, and then will focus on considerations and perspectives related to the production of MCR in a heterologous host. For more detailed reviews on MCR biochemistry, the reader is referred to recent excellent reviews by Thauer (2019) and Ragsdale et al. (2017).

## Overview of Methanogenesis

Methanogenic archaea (“methanogens”) are ancient and diverse microorganisms within the archaeal domain of life (Battistuzzi et al., 2004; Adam et al., 2017). They are found in a wide range of anaerobic environments including marine and freshwater habitats, anoxic soils, and as important components of animal microbiomes (Moissl-Eichinger et al., 2018; Lyu et al., 2018a; Borrel et al., 2020). As their sole source of energy, methanogens carry out a form of anaerobic respiration known as methanogenesis, which reduces simple oxidized carbon compounds to generate methane as an end product. There are three main types of methanogenic metabolism depending on the substrate used for methanogenesis (Liu and Whitman, 2008; Costa and Leigh, 2014; Yan and Ferry, 2018). Hydrogenotrophic methanogenesis involves the reduction of CO_2_ to CH_4_, usually with H_2_ as the electron donor. Some hydrogenotrophic methanogens are also able to use other electron donors, such as formate, CO, alcohols, and iron (Dolfing et al., 2008; Ferry, 2010; Kurth et al., 2020). Methylotrophic methanogenesis involves the activation of methylated compounds, such as methanol and trimethylamine *via* substrate-specific corrinoid proteins, which then transfer the methyl group into methanogenesis *via* CH_3_-S-CoM. The more recently discovered methoxydotrophic pathway involves methanogenesis from methoxylated aromatic compounds (Mayumi et al., 2016), where the methyl group is transferred to tetrahydromethanopterin instead of coenzyme M (HS-CoM; Kurth et al., 2021a). Finally, acetoclastic methanogenesis utilizes acetate as a methanogenesis substrate, where the carboxyl group is oxidized to CO_2_ and the methyl group is reduced to CH_4_. Although acetoclastic methanogenesis is the least bioenergetically favorable, 2/3 of biologically derived methane comes from acetate (Lyu et al., 2018a). While there are notable distinctions across the three methanogenic pathways, the key methane-generating step is always catalyzed by MCR (Figure 1).

Methanogenesis produces nearly a billion tons of methane each year, which accounts for at least 70% of global methane emissions (Conrad, 2009; Kirschke et al., 2013; Jackson et al., 2020). About half of this methane is consumed by methanotrophic microorganisms, while the remainder unfortunately escapes to our atmosphere (Kirschke et al., 2013). Although much less abundant compared to CO_2_, methane is a more potent greenhouse gas since it has at least a 25-fold higher global warming potential than CO_2_ over a 100-year period (Montzka et al., 2011). The rising methane concentration is believed to account for ~20% of the current global warming trend (Lyu et al., 2018a). Thus, the development of strategies to curb methane emissions is essential to mitigate climate change. Indeed, MCR is a highly pursued target for developing inhibitors toward biological methane production (Duin et al., 2016; Yu et al., 2021).

## Overview of the Anaerobic Oxidation of Methane

Anaerobic methanotrophic archaea (ANME) are related to methanogens and are capable of oxidizing methane in the absence of O_2_, consuming substantial amounts of methane in anaerobic environments and thus playing a critical role in the global methane budget (Knittel and Boetius, 2009). Metagenome and gene/protein expression data have revealed that ANME contain and express previously characterized methanogenic genes, indicating that they utilize a reverse methanogenesis pathway to oxidize methane to CO_2_ (Hallam et al., 2004; Meyerdierks et al., 2010; Stokke et al., 2012; Timmers et al., 2017). Most commonly, ANME exist with syntrophic sulfate-reducing bacteria that allow AOM to be coupled with sulfate reduction (Nauhaus et al., 2002; Knittel and Boetius, 2009; Holler et al., 2011; Wegener et al., 2016). In these consortia, ANME carry out the oxidation reactions, with MCR presumably catalyzing the initial methane activation step (Scheller et al., 2010; Figure 1). The reducing equivalents generated throughout methane oxidation to CO_2_ are transferred to the bacteria, likely mediated by multi-heme *c-*type cytochromes, for use in sulfate reduction (McGlynn et al., 2015; Wegener et al., 2015, 2016). Additionally, single archaeal populations have been identified that have the genes necessary for performing AOM as well as sulfite reduction (McKay et al., 2019) or nitrate/nitrite reduction (Haroon et al., 2013), or may transfer electrons directly to metals (He et al., 2018), indicating that there may be exceptions to the paradigm of interspecies redox coupling. On the basis of metagenomic data, ANME are separated into four main clades: ANME-1 (Hinrichs et al., 1999), ANME-2 (Orphan et al., 2001, 2002), ANME-2d (Raghoebarsing et al., 2006; more recently referred to as *Ca.* Methanoperedenaceae; Haroon et al., 2013), and ANME-3 (Knittel et al., 2005; Niemann et al., 2006).

## Overview of Methyl-Coenzyme M Reductase

**Figure 2:**
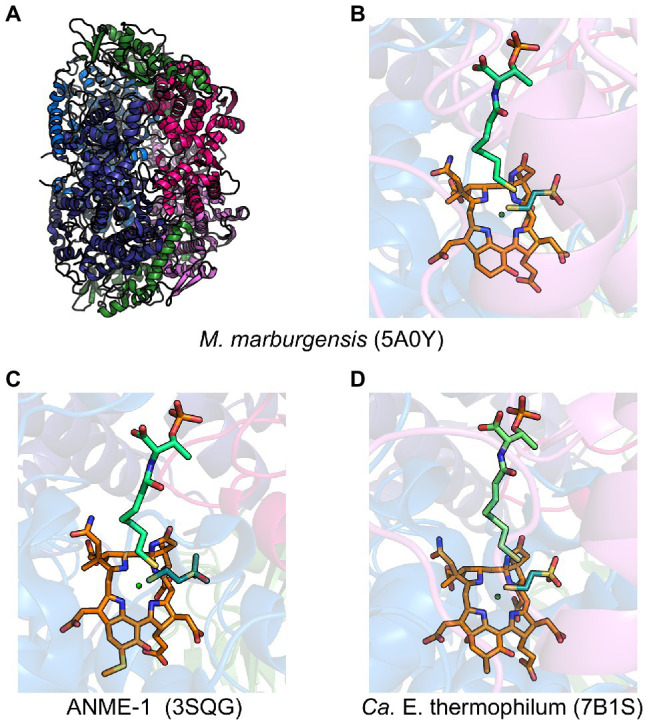
Representative crystal structures of MCRs and ECR. **(A)** MCR from *M. marburgensis* and **(B)** the associated active site with F_430_, HS-CoB, and HS-CoM. **(C)** Black Sea mat ANME-1 MCR active site with 17^2^-methylthio-F_430_, HS-CoB, and HS-CoM. **(D)** *Ca.* E. thermophilum ECR active site with dimethyl-F_430_, HS-CoB, and HS-CoM. α subunits are shown in marine and deep blue, *β* subunits in hot pink and violet, γ subunits in forest green, F_430_ in orange, coenzyme M in deep teal, and coenzyme B in lime green.

MCR is a dimer of heterotrimers with a *α*_2_*β*_2_*γ*_2_ configuration (Figure 2A), harboring two active sites that are only accessible through a 50 Å channel (Ermler et al., 1997). Each active site contains the nickel hydrocorphin prosthetic group, coenzyme F_430_ (Ellefson et al., 1982, Pfaltz et al., 1982, Livingston et al., 1984, Farber et al., 1991; Figures 1 and 2). The active form of MCR contains F_430_ in the Ni(I) oxidation state (Goubeaud et al., 1997). In the methane-forming direction, MCR catalyzes the conversion of CH_3_-S-CoM and HS-CoB to methane and the CoM-S-S-CoB heterodisulfide (Figure 1). Only one site is activated at any given time (“half-of-the-sites reactivity”), and thus the two active sites are proposed to function similar to a two-stroke engine where binding of the substrates in one active site induces a conformational change that provides energy for the heterodisulfide product to be expelled in the other active site (Goenrich et al., 2005; Scheller et al., 2013). Although any ANME MCR has yet to be enzymatically investigated *in vitro*, MCR from *M. marburgensis* can catalyze the reverse methane oxidation reaction at rates comparable to those measured in AOM consortia *in vivo* (Scheller et al., 2010), supporting the proposal that ANME utilize MCR to oxidize methane.

Recent mechanistic studies have provided evidence that the reaction occurs by “mechanism II,” involving a methyl radical intermediate (Wongnate et al., 2016) that was originally proposed on the basis of quantum mechanical modeling studies (Pelmenschikov et al., 2002; Pelmenschikov and Siegbahn, 2003; Chen et al., 2012, 2014). The major alternative mechanism (“mechanism I”) involves nucleophilic chemistry with a Ni(III)-methyl intermediate (Yang et al., 2007; Dey et al., 2010). In the proposed radical mechanism, Ni(I) induces homolytic cleavage of the methyl-sulfur bond of CH_3_-S-CoM to generate a methyl radical and Ni(II). The methyl radical then reacts with HS-CoB to produce methane and a •S-CoB radical, which reacts with the Ni-bound CoM thiolate to generate a disulfide anion radical. One-electron transfer to Ni(II) then releases the heterodisulfide and regenerates the Ni(I) (Wongnate et al., 2016).

## MCR Structures and Post-translational Modifications

The structures of MCR for which crystal structures have been obtained from various methanogens and alkane-oxidizing organisms are all remarkably similar. Based on phylogenetic and structural comparison, MCRs from *Methanobacteriales* and *Methanococcales* were classified into MCR types I, II, and III (Wagner et al., 2017). The different MCR types have representative crystal structures and mainly differ in their electrostatic surface potentials, loop architectures, and the C-terminal end of their γ-subunits that interact with α and β subunits. Based on phylogenetic comparisons, MCRs from other organisms, such as *Methanomicrobiales, Methanosarcinales, Methanocellales,* and ANME-1, are distinct from the defined types I-III present in *Methanobacteriales* and *Methanococcales* (Wagner et al., 2017).

MCR crystal structures with HS-CoB and the substrate analog HS-CoM show the sulfhydryl group of HS-CoM serving as an axial ligand to the Ni(II) of F_430_ (Figure 2B). However, all MCR crystal structures are of the enzyme in its inactive Ni(II) state or a chemically modified methyl-Ni(III) state (Cedervall et al., 2011). Thus, the true coordination state of Ni(I) and the binding conformation of CH_3_-S-CoM remains unclear since a crystal structure with CH_3_-S-CoM has never been obtained. However, recent studies (Patwardhan et al., 2021) have provided new evidence for the possible orientation of CH_3_-S-CoM binding to the nickel center of F_430_. Interestingly, results indicate that there is no nickel-sulfur interaction and thus suggest that the thioether portion of the substrate does not bind to the Ni(I) (Patwardhan et al., 2021). Instead, CH_3_-S-CoM appears to bind to Ni(I) through the sulfonate group. This proposed alternate binding scenario puts the reactive portions of the two substrates in close proximity so that the subsequently generated proposed methyl radical is in position to abstract a hydrogen atom from HS-CoB (Patwardhan et al., 2021).

**Figure 3:**
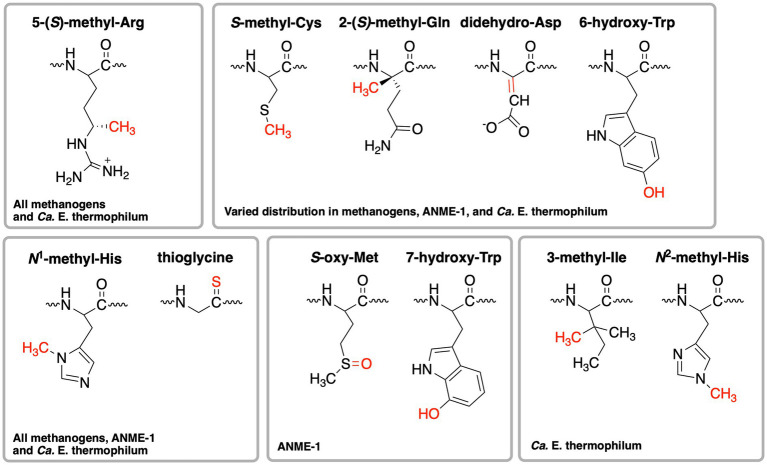
Structures of post-translational modifications identified in the active sites of various MCRs and ECR. The distribution of these PTMs is further summarized in Table 1.

When the first crystal structure of MCR from *M. marburgensis* (Figures 2A,B) was solved, five unusual post-translational modifications (PTMs) were revealed in the *α* subunit near the active site (Ermler et al., 1997). Since then, subsequent work has discovered additional MCR PTMs, where the presence of specific PTMs varies depending on the organism (Table 1; Grabarse et al., 2000; Kahnt et al., 2007; Wagner et al., 2016; Wagner et al., 2017; Kurth et al., 2021b). PTMs found in methanogens include three strictly conserved modifications—*N*^1^-methylhistidine, 5-(*S*)-methylarginine, and thioglycine—as well as a handful of more variable modifications including *S*-methylcysteine, 2-(*S*)-methylglutamine, didehydroaspartate, and 6-hydroxytryptophan (Table 1 and Figure 3). The impacts of these PTMs remain a major area of research in the field as their precise role in catalysis and/or active site structure remains unclear. MCR PTMs were recently summarized in a mini-review (Chen et al., 2020), but we will outline key aspects here.

**Table 1:** Summary of MCR crystal structures with associated PTM content.

MCR crystal structure	PDB	*N*^1^-methyl-His	*S*-methyl-Cys	2-(*S*)-methyl-Gln	5-(*S*)-methyl-Arg	Thioglycine	Didehydro-Asp	6-hydroxy-Trp	7-hydroxy-Trp	3-methyl-Ile	*N*^2^-methyl-His	*S*-oxy-Met
*Methanothermobacter marburgensis* MCR I (Ermler et al., 1997; Wagner et al., 2016)	5A0Y	+	+	+	+	+	+	−	−	−	−	−
*Methanothermobacter marburgensis* MCR II (Wagner et al., 2016)	5A8R	+	+	+	+	+	+	−	−	−	−	−
*Methanosarcina barkeri* (Grabarse et al., 2000; Wagner et al., 2016)	1E6Y	+	+	−	+	+	+	−	−	−	−	−
*Methanosarcina acetivorans* (Nayak et al., 2020)		+	+	−	+	+	+	−	−	−	−	−
*Methanopyrus kandleri* (Grabarse et al., 2000; Kahnt et al., 2007)	1E6V	+	−	+	+	+	−	−	−	−	−	−
*Methanotorris formicicus* (Wagner et al., 2017)	5N2A	+	−	+	+	+	−	+	−	−	−	−
*Methanothermobacter wolfeii* (Wagner et al., 2016)	5A8K	+	+	+	+	+	−	−	−	−	−	−
*Methanothermococcus thermolithotrophicus* (Wagner et al., 2017)	5N1Q	+	−	+	+	+	−	−	−	−	−	−
*Methermicoccus shengliensis* (Kurth et al., 2021b)	7NKG	+	−	−	+	+	−	−	−	−	−	−
ANME–1 from Black Sea mats (Shima et al., 2012)	3SQG	+	+/−^a^	−	−	+/−^a^	−	−	+	−	−	+
*Ca.* Ethanoperedens thermophilum (Hahn et al., 2021)	7B1S	+	+	+	+	+	−	−	−	+	+	−

a Early mass spectrometry data indicated that ANME-1 MCR lacked the *S*-methylcysteine as well as thioglycine (Kahnt et al., 2007), while the ANME-1 MCR crystal structure showed the thioglycine was present (Shima et al., 2012). However, the sample used for crystallization represented a mixed population where 30% contained thioglycine but not *S*-methylcysteine, while the majority (70%) contained *S*-methylcysteine but not thioglycine and did not result in crystal formation (Shima et al., 2012). (+) indicates PTM is present and (−) indicates PTM is absent.

Significant progress has been made toward identifying the enzymes involved in installing MCR PTMs, including identification of the radical *S*-adenosylmethionine (SAM) enzyme catalyzing the difficult methylation reaction to produce 5-(*S*)-methylarginine (Deobald et al., 2018; Radle et al., 2019; Lyu et al., 2020) and identification of the enzymes responsible for the thioglycine transformation (Nayak et al., 2017). Both of these PTMs appear to at least be important for the stability of MCR from *Methanosarcina acetivorans*, especially under thermal stress (Nayak et al., 2017, 2020; Deobald et al., 2018). The methylated arginine seems to have a more significant impact on *Methanococcus maripaludis* MCR, where a deletion strain lacking this modification showed a highly impaired growth rate and the rate of methanogenesis was only about half the rate of wild type (Lyu et al., 2020). Most recently, the methyltransferase necessary for the synthesis of *S*-methylcysteine was identified (Nayak et al., 2020). Through the production of a *M. acetivorans* deletion strain lacking the genes involved in 5-(*S*)-methylarginine, thioglycine, and *S*-methylcysteine biosynthesis, the associated MCR variant was produced and its crystal structure was solved, which was surprisingly indistinguishable from the wild-type structure (Nayak et al., 2020). Growth studies with the associated deletion strain suggested that epistatic interactions among MCR PTMs influence the stability and *in vivo* activity of the enzyme (Nayak et al., 2020). However, *in vitro* kinetic studies have not yet been carried out on MCR variants lacking one or more PTMs, which will be required to make any conclusions about the specific functions and importance of the respective PTMs.

**Figure 4:**
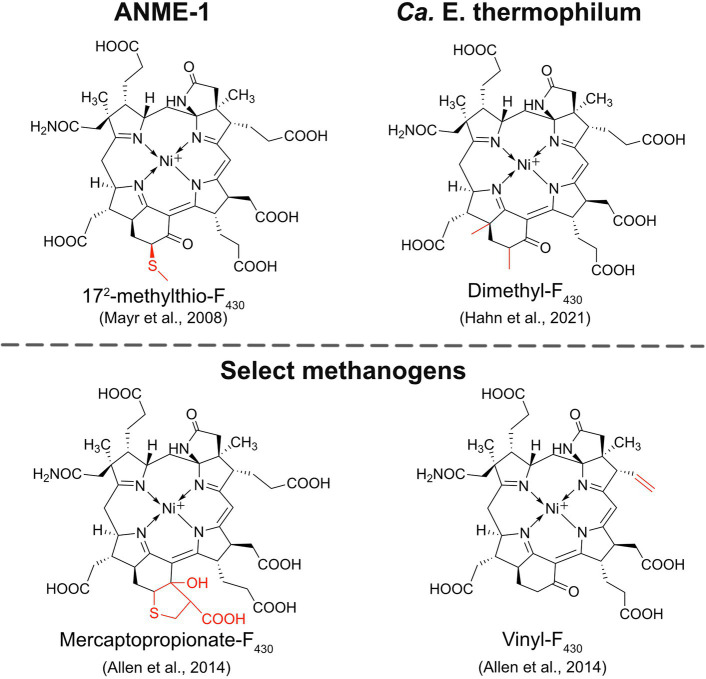
Structures of modified F_430_s. The structures of the modified F_430_s in ANME-1 and *Ca.* *E. thermophilum* are confirmed based on NMR and/or crystal structures while the modifications in select methanogens are proposed based on mass spectrometry data.

ANME-1 is the only ANME clade for which an MCR crystal structure has been obtained. By purifying and crystallizing the enzyme directly from a Black Sea mat sample, the crystal structure of the ANME-1 MCR was solved to 2.1 Å resolution (Shima et al., 2012). This ANME MCR possesses the same overall structure as methanogenic MCRs. In particular, the active site channel seems be strictly conserved in both ANME-1 and methanogens, with HS-CoM and HS-CoB holding virtually the same position and conformation (Figure 2C). This result excludes the possibility of ANME MCR using different substrates/products, further supporting that methane oxidation catalyzed by MCR in ANME is the reverse of the methane-generating step in methanogens (Figure 1). The ANME-1 MCR structure does possess a few notable differences. First, the active site contains a modified F_430_, 17^2^-methylthio F_430_ (Figures 2C, 4), which was previously structurally characterized by mass spectrometry and NMR (Mayr et al., 2008). This modified F_430_ appears to be accommodated by the replacement of the bulky 2-(*S*)-methylglutamine found in methanogens with Val419 in ANME-1. Second, ANME-1 MCR contains five distinct cysteines between F_430_ and the protein surface, suggesting a potential redox-relay system that could be used to reduce F_430_ to the active Ni(I) state (Shima et al., 2012). Third, PTM patterns vary between ANME-1 MCR and methanogenic MCR. ANME-1 does not contain the highly conserved arginine methylation seen in methanogens, however, ANME-1 MCR does contain two unique PTMs, 7-hydroxytryptophan and *S*-oxymethionine. Also present in ANME-1 MCR are *N*^1^-methylhistidine and thioglycine (Shima et al., 2012; Table 1 and Figure 3).

Very recently, the crystal structure of the MCR homolog from an anaerobic ethane oxidizing archaeon, *Ca.* Ethanoperedens thermophilum, was determined (Hahn et al., 2021). This enzyme apparently does not take other alkane substrates outside of ethane (Hahn et al., 2020) and thus has been designated as ethyl-coenzyme M reductase (ECR; Hahn et al., 2021). Although ECR follows the overall structural trend of known MCRs, there are notable differences to consider. First, ECR is 20 kDa larger than canonical MCRs, mainly due to three insertions in the α subunit, one insertion in the β subunit, and one insertion in the γ subunit. These insertions impact surface charges and contribute to the unique architecture of the ethane tunnel, which provides a 33 Å hydrophobic path to the active site (Hahn et al., 2021). The tunnel is flanked by post-translationally modified amino acids, including two unique PTMs—*N*^2^-methylhistidine and 3-methylisoleucine (Table 1 and Figure 3). Notably, the ECR structure revealed that the F_430_ nickel is coordinated by methionine rather than the canonical glutamine, and a modified version of F_430_ with two methyl groups is present (dimethyl-F_430_; Figures 2D, 4). Additionally, an active site loop (α367-374) contains a tryptophan residue (αTrp^373^) instead of the canonical phenylalanine found in other MCRs. This loop shifts the position of F_430_, which is stabilized by hydrogen bonds with αAsn^375^ along with a clamping effect from αTyr^376^ and αPhe^441^, resulting in a 11.4° tilt on the porphinoid ring. Consequently, the active site volume is increased to adequately accommodate ethane. The authors propose that the F_430_ methylations likely serve to maintain the structure and reactivity of the cofactor in the expanded active site (Hahn et al., 2021).

## Modified F_430_ Coenzymes

An interesting and underexplored area in the MCR field is the potential functions and importance of F_430_ modifications. F_430_ is only known to function with MCRs and ACRs, indicating that nature has evolved a specialized coenzyme to catalyze the difficult reactions of methane formation and methane/alkane activation. The first modified F_430_ to be discovered was 17^2^-methylthio-F_430_ (Figure 4), which was originally identified and structurally characterized from Black Sea mat samples enriched with ANME-1 (Mayr et al., 2008). This modified F_430_ is presumed to be the primary physiologically active version of F_430_ in ANME-1 since it was later characterized in the crystal structure of ANME-1 MCR (Shima et al., 2012; Figure 2C). ANME-2 organisms apparently do not contain 17^2^-methylthio-F_430_ (Mayr et al., 2008; Kaneko et al., 2014), and the potential F_430_ modifications present in ANME-3 have not yet been reported to our knowledge. In 2014, additional F_430_ modifications were identified in select *Methanococcales* methanogens (Allen et al., 2014). Mercaptopropionate-F_430_, containing a cyclized mercaptopropionate moiety bound as a thioether, was identified in *Methanocaldococcus jannaschii* and *Methanococcus maripaludis*, while vinyl-F_430_ was observed in *M. maripaludis* and *Methanococcus vannielii* (Figure 4). These structures were proposed based on mass spectrometry data, and thus still need to be confirmed by NMR or crystallography. Finally, most recently, the dimethyl-F_430_ present in ECR was discovered (Hahn et al., 2021; discussed above; Figure 4). One important aspect to note is that the F_430_ modifications in methanogens are not always present and the modifications generally exist as a minor component compared to the unmodified F_430_ (Allen et al., 2014). The situation appears to be different in ANME-1, where 17^2^-methylthio-F_430_ is the predominant form (Kaneko et al., 2014). Additionally, for both ANME-1 and *Ca.* E. thermophilum, 17^2^-methylthio-F_430_, and dimethyl-F_430_ are confirmed to function with the respective MCR/ECR since they were identified in the crystal structures (Shima et al., 2012; Hahn et al., 2021). In contrast, the modified F_430_s in methanogens have only so far been identified in small molecule cell extracts and have not been observed in any methanogen MCR crystal structures.

Understanding how modified F_430_s affect MCR will be important for the design of optimized heterologous systems for recombinant MCR production, especially for anaerobic methane/alkane oxidation applications. It will also be necessary to identify the enzymes involved in their biosynthesis. The complete biosynthetic pathway for F_430_ has been described (Zheng et al., 2016; Moore et al., 2017), but the enzymes required for the installation of F_430_ modifications are currently unknown.

## MCR Operon Organization

**Figure 5:**
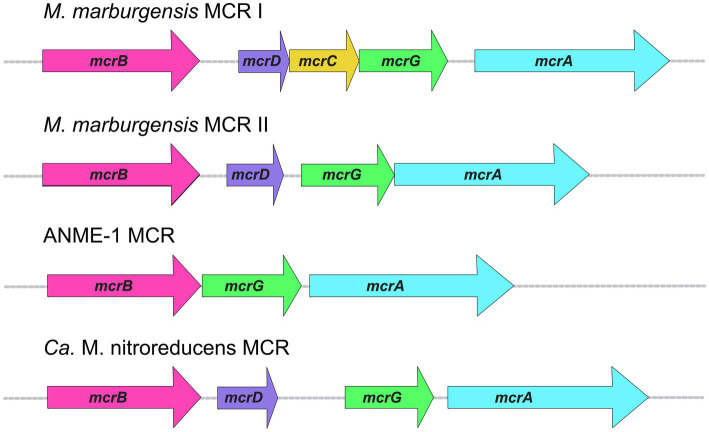
MCR operon organization in selected organisms.

The three MCR subunits are encoded by *mcrA*, *mcrB*, and *mcrG*, which are usually present in an MCR operon along with two other genes (*mcrD* and *mcrC*). The most common MCR operon across all methanogens is *mcrBDCGA* (Figure 5). McrD and McrC are accessory proteins whose functions have yet to be confirmed. McrC was a component of the large complex of proteins identified as being responsible for the reduction of F_430_ to its Ni(I) active state; thus McrC likely plays a role in MCR activation (Prakash et al., 2014). McrD may serve as a chaperone protein that binds F_430_ for subsequent delivery to the MCR active site (Zheng et al., 2016). Early studies demonstrated that McrD interacts with MCR (Sherf and Reeve, 1990) and it also co-purified with a recombinant MCR expressed in *M. maripaludis* (Lyu et al., 2018b). Finally, McrD was shown to alleviate product inhibition in the final step of F_430_ biosynthesis *in vitro*, presumably due to its ability to bind the newly synthesized F_430_ coenzyme (Zheng et al., 2016).

An interesting aspect of MCR operons is the variability in the presence of accessory proteins, or the presence of additional operons. In some methanogens, including *M. marburgensis*, there is a second MCR operon that encodes for MCR isozyme II (Rospert et al., 1990). Most work on MCR has been performed on MCR isozyme I, whose operon in *M. marburgensis* is the typical *mcrBDCGA* operon. Less studied is MCR II, whose operon lacks *mcrC* (Figure 5). The two isozymes are highly similar in sequence as well as overall structure and active site architecture (Wagner et al., 2016), but some notable differences have been reported. MCR I and MCR II show significant differences in electrostatic surface potentials, which allows for convenient separation of the two isozymes *via* anion exchange chromatography (Rospert et al., 1990; Duin et al., 2011). Because MCR II was purified in larger quantities from cells in log phase, and MCR I was purified in larger quantities from cells at the end of growth, it was concluded that MCR II is expressed in non-gas-limiting conditions, while MCR I is expressed during gas-limiting conditions (Rospert et al., 1990; Bonacker et al., 1992). Further studies on both isoenzymes revealed that MCR I and MCR II expression is affected by pH, temperature, and availability of both H_2_ and CO_2_ (Bonacker et al., 1992), and *in vitro* kinetic studies revealed differing catalytic properties (Bonacker et al., 1993).

In addition to *M. marburgensis*, many other members of *Methanobacteriales*, and some members of *Methanococcales* and *Methanomicrobiales* contain two MCR isozymes. The presence of two MCR isozymes may present a possible evolutionary advantage which could allow methanogens to express different versions of MCR when faced with changes in environmental conditions, such as substrate limitation. The fact that the MCR II gene cluster lacks *mcrC* would indicate that either McrC is not necessary for the function of MCR II or that the McrC encoded in the MCR I gene cluster can be utilized.

The difference between MCR I and MCR II gene clusters is not the only instance in which the MCR operon displays variability. The MCR operon from ANME-1 isolated from a Black Sea mat lacks both *mcrD* and *mcrC* (Meyerdierks et al., 2010; Figure 5). A McrC homolog is present outside of the MCR operon in ANME-1 (BSM_08630, 51% identity to McrC from *M. marburgensis*), but McrD appears to be completely absent. The ANME-2d organism, *Ca.* Methanoperedens nitroreducens, contains an MCR operon like that of MCR II in *M. marburgensis*, where only *mcrD* is present without *mcrC* (Haroon et al., 2013; Figure 5). Similar to ANME-1, a likely *mcrC* exists outside of the MCR operon (ANME2D_00875, 53% identity to McrC from *M. marburgensis,* 50% identity to McrC from ANME-1 mentioned above). This poses interesting questions regarding the high conservation of the primary MCR I operon *mcrBDCGA* across different methanogenic species, whereas ANME MCR operons lack one or more of the accessory proteins in the operon. Additionally, ANME-1 organisms seem to completely lack *mcrD*. A better understanding of the functions and specificity of these and potentially additional yet to be discovered accessory proteins is crucial for the development of optimized recombinant MCR expression systems.

## Alkyl-Coenzyme M Reductases

MCR variants known as alkyl-coenzyme M reductases (ACRs) carry out the anaerobic oxidation of various non-methane alkane substrates. The first report of ACR-dependent anaerobic oxidation of an alkane other than methane was published in 2016 when members of the GoM-Arch87 clade, closely related to *Methanosarcinales*, were proposed to carry out the anaerobic oxidation of butane to CO_2_ (Laso-Pérez et al., 2016). Similar to ANME-1, these organisms form consortia with HotSeep-1 sulfate-reducing bacteria and couple butane oxidation to sulfate reduction. Two archaeal genomes were assembled from the butane enrichment cultures that resulted in the proposed names for two new organisms, *Ca.* Syntrophoarchaeum butanivorans and *Ca.* Syntrophoarchaeum caldarius (Laso-Pérez et al., 2016). Intriguingly, these organisms contain four different *mcrBGA* genes clusters—three of the four ACR gene sets are arranged in operons in *Ca.* S. butanivorans, while all four are arranged in operons in *Ca.* S. caldarius. The butane-dependent formation of butyl-S-CoM was confirmed in these cultures, suggesting that butane oxidation involves the use of the MCR homolog(s). The GoM-Arch87 enrichment cultures were also tested with other hydrocarbons and, interestingly, propane enriched cultures resulted in propane dependent sulfate reduction. As with the butane cultures, propyl-S-CoM was detected, indicating the involvement of an ACR in propane oxidation. Other alkane oxidizers include *Ca.* Argoarchaeum ethanivorans (Chen et al., 2019) and *Ca.* E. thermophilum (Hahn et al., 2020), archaeal species that activate ethane using ECR (discussed in MCR structures and post-translational modifications section) to form ethyl-S-CoM, which is subsequently oxidized to CO_2_. These ethane oxidizers only utilize ethane and cannot metabolize other alkanes.

In addition to the involvement of ACRs in short-chain alkane oxidation, recently published work suggests the use of ACRs to activate long-chain alkanes in oil reservoirs (Zhou et al., 2022). *Ca.* Methanoliparum couples the degradation of long-chain alkanes to methanogenesis in a process that is independent of syntrophic partners. Metagenomic and transcriptomic data indicated the presence of several different species, which contain and expresses genes encoding putative ACRs as well as MCRs. Additionally, the presence of hexadecyl-S-CoM and other long-chain alkane R-S-CoM derivatives were confirmed by mass spectrometry, thus indicating the involvement of an ACR in alkane activation (Zhou et al., 2022).

## Other Divergent MCRs

Traditionally, all methanogens were thought to belong to the Euryarchaeota phylum, however, this definition was challenged with the discovery of putative methane metabolism in the Bathyarchaeota phylum (Evans et al., 2015). Metagenome data revealed the presence of *mcrABG* genes and putatively *mcrCD*, as well as several other genes for methylotrophic methanogenesis. The MCR primary sequence and predicted structure analysis showed putative binding sites for HS-CoM, HS-CoB, and F_430_, suggesting that the enzyme utilizes the same substrates and coenzyme (Evans et al., 2015). In addition to Bathyarchaeota, five metagenomes from a proposed new archaeal phylum termed *Ca.* Vestraetearchaeota were shown to contain divergent *mcrA* sequences (Vanwonterghem et al., 2016). Metabolic reconstructions of the assembled genomes revealed the presence of key genes associated with methylotrophic methanogenesis, including a complete *mcrBDCGA* operon. It is hypothesized that these organisms would perform H_2_-dependent methylotrophic methanogenesis. However, it is still unclear if this new phylum is comprised of organisms that perform methanogenesis as a preferential metabolism since the assembled genomes in this study also showed that these organisms likely have the capacity to perform fermentative metabolism (Vanwonterghem et al., 2016).

Archaeoglobi is a class of thermophilic organisms within the Euryarchaeota that were generally believed to be non-methanogenic (Bapteste et al., 2005; Hartzell and Reed, 2006; Boyd et al., 2019). This is because complete MCR-encoding genes and methyl-H_4_M(S)PT:coenzyme M methyltransferase (MTR) *MtrABCDEFGH* complex genes had never been identified, even though other characteristic hydrogenotrophic methanogenesis genes as well as archaeal type Wood-Ljungdahl pathway genes have been identified in some Archaeoglobi genomes (Klenk et al., 1997; Bapteste et al., 2005). However, recent metagenome data have demonstrated that some Archaeoglobi contain genes encoding MCR. *Ca.* Polytropus marinifundus contains two divergent *mcrABG* operons similar to Bathyarchaeota and Syntrophoarchaeum as well as other potential genes for alkanotrophic metabolism (Boyd et al., 2019). Another new genus of Archaeoglobi, *Ca.* Methanomixophus, contains MTR complex genes as well as a complete *mcrBDCGA* operon, along with predicted ligand binding sites for HS-CoM, HS-CoB, and F_430_ (Liu et al., 2020). Metatranscriptomic experiments showed active hydrogen-dependent methylotrophic methanogenesis as well as heterotrophic fermentation. Additionally, one of the new proposed organisms, *Ca.* Methanomixophus hydrogenotrophicum, possesses the genes to conserve energy *via* AOM coupled to syntrophic sulfate reduction, while *Ca.* Methanomixophus dulitatem contains its own sulfate reduction genes that would allow for a methane oxidizing lifestyle (Liu et al., 2020).

## Methanogens as Hosts for the Heterologous Production of Recombinant MCRs

The recent and continuing discoveries of diverse putative MCRs and ACRs highlight the need to develop effective tools to study the catalytic capabilities of these enzymes with likely very different enzymatic properties. Additionally, MCR is a highly attractive, yet challenging, target for potential use in bioengineering applications for biofuel production, either for methane generation or methane/alkane conversion applications (Conrado and Gonzalez, 2014; Haynes and Gonzalez, 2014). Since ANME utilize MCR in the methane oxidation direction, ANME MCRs are especially appealing biocatalysts for potentially converting abundant methane reserves into more usable liquid fuels and other value-added chemicals (Mueller et al., 2015; Lawton and Rosenzweig, 2016a,b). However, as mentioned previously, no ANME MCR has been studied *in vitro* and thus it is unclear whether ANME MCRs will be better suited for this purpose compared to methanogenic MCRs.

Since AOM consortia grow slowly, with doubling times on the month timescale (Laso-Pérez et al., 2018; Bhattarai et al., 2019), and to low cell densities, obtaining enough cells to purify the native MCR from ANME organisms in sufficient quantities for kinetic and mechanistic studies is not very feasible. Additionally, ANME organisms are not yet genetically tractable and thus are not amenable to bioengineering applications. Thus, the development of heterologous expression systems for ANME MCRs as well as other diverse MCRs from unculturable or difficult-to-culture archaea would be highly advantageous. Traditional hosts, such as *Escherichia coli*, seem to pose currently unsurmountable challenges to achieve this goal since they do not possess the biochemical machinery for F_430_ biosynthesis, post-translational modifications, or MCR assembly and activation. Thus, the current likely best option for a heterologous host is a fast-growing methanogen for which genetic manipulation methods exist. The two highly studied model methanogens for which well-established and robust genetic tools exist are *Methanosarcina acetivorans* and *Methanococcus maripaludis*. The following paragraphs and Table 2 summarize the major available genetic tools in these organisms as well as recently described genetic tools in *Methanocaldococcus jannaschii* and *Methanothermobacter thermautotrophicus.*

**Table 2:** Summary of major genetic tools available in methanogens with associated references.

	*Methanosarcina acetivorans/barkeri*	*Methanosarcina mazei*	*Methanococcus maripaludis* (S2 and JJ)	*Methanocaldococcus jannaschii*	*Methanothermobacter thermautotrophicus* Δ*H*
Transformation methods	Liposome mediated (Metcalf et al., 1997; Buan et al., 2011)	Liposome mediated (Metcalf et al., 1997; Ehlers et al., 2005)	Polyethylene glycol mediated (Tumbula et al., 1994; Sarmiento et al., 2011; Natural competence; Fonseca et al., 2020)	Heat shock (Susanti et al., 2019)	Interdomain conjugation (Fink et al., 2021)
Shuttle vectors	Metcalf et al., 1997; Buan et al., 2011	Metcalf et al., 1997	Whitman et al., 1997; Sarmiento et al., 2011	–	Fink et al., 2021
Positive selection marker	Puromycin (Metcalf et al., 1997; Buan et al., 2011)	Puromycin (Metcalf et al., 1997; Ehlers et al., 2005) Neomycin (Mondorf et al., 2012)	Puromycin and neomycin (Whitman et al., 1997; Sarmiento et al., 2011)	Mevinolin and Simvastatin (Susanti et al., 2019)	Neomycin (Fink et al., 2021)
Counterselection marker	*hpt* (8-azahypoxanthine) (Pritchett et al., 2004; Buan et al., 2011)	*hpt* (8-azahypoxanthine) (Ehlers et al., 2011)	*hpt* (8-azahypoxanthine), *upt* (6-azauracil) (Moore and Leigh, 2005; Sarmiento et al., 2011)	–	–
Markerless genetic exchange	Pritchett et al., 2004; Buan et al., 2011	Ehlers et al., 2011	(Moore and Leigh, 2005; Sarmiento et al., 2011	–	–
Inducible promoters	Tetracycline-inducible promoter (Guss et al., 2008) Acetate regulated promoter (Macauley et al., 2009)	Trimethylamine regulated promoter (Mondorf et al., 2012)	*Nif* promoter (Lie and Leigh, 2002; Chaban et al., 2007) Phosphate sensing promoter (Akinyemi et al., 2021)	–	–
CRISPR/Cas System	CRISPR/Cas9 (Nayak and Metcalf, 2017)	–	CRISPR/Cas12 (Bao and Scheller, 2021)	–	–

*Methanosarcina* species are cytochrome-containing methanogens that are capable of using the widest variety of methanogenic substrates compared to other genera. Depending on the growth substrate, *M. acetivorans* exhibits doubling times as low as 8 h at 37°C. Compared to other methanogens with available genetic tools, *Methanosarcina* are phylogenetically most closely related to ANME (Knittel et al., 2005), thus making *Methanosarcina* the most logical choice as potential heterologous hosts for ANME MCRs. *M. acetivorans* C2A is most commonly utilized for genetic experiments, but *Methanosarcina barkeri* Fusaro is also used successfully and the same basic tools have been developed for both species (Buan et al., 2011). Both organisms are mesophilic and require that they are maintained in high-salt medium to prevent cells from growing in clumps, which can pose issues for genetic experiments. Routine genetic experiments normally involve a parental strain containing a deletion of the hypoxanthine phosphoribosyltransferase *hpt* gene that is used as a counterselection marker (Pritchett et al., 2004), and often also contain a ΦC31 *attP* site inserted at the *hpt* locus, allowing for insertion of plasmids containing the complementary *attB* sequence into the host chromosome *via* recombination (Guss et al., 2008). A comprehensive description of the different plasmids and strains of *Methanosarcina* that have been used for various purposes, including gene deletions and recombinant expression, has been reported (Buan et al., 2011). Transforming *Methanosarcina* species involves the well-established liposome-mediated transformation, where the transformation efficiency is as high as 20% in *M. acetivorans*, but is substantially lower in other *Methanosarcina* species (Metcalf et al., 1997). For inducible expression of recombinant proteins in *Methanosarcina*, the tetracycline-inducible system can be used, which allows for the expression of proteins at a desired time point during growth (Guss et al., 2008). The *cdh* operon promoter has also been used successfully to drive acetate-dependent overexpression of a carbonic anhydrase in *M. acetivorans* (Macauley et al., 2009). More recent work has generated a new series of suicide plasmids that simplify the cloning process and allow for facile expression and purification of tagged proteins in *Methanosarcina* (Shea et al., 2016). Additionally, an efficient CRISPR/Cas9 system has been developed for *M. acetivorans* (Nayak and Metcalf, 2017). Notably, this system has been used to add a tandem affinity purification tag to the N-terminus of the native McrG gene in *M. acetivorans*, thus greatly facilitating MCR purification (Nayak and Metcalf, 2018). Finally, genetic tools have also been utilized for *Methanosarcina mazei* (Ehlers et al., 2005), including markerless genetic exchange using similar methods developed for *M. acetivorans* and *M. barkeri* (Pritchett et al., 2004; Ehlers et al., 2011). A trimethylamine inducible promoter has also been used to successfully heterologously overexpress a fusion-tagged protein in *M. mazei* (Mondorf et al., 2012).

*Methanococcus maripaludis* is a hydrogenotrophic mesophilic methanogen with a relatively fast doubling time of 2 h at 37°C (Whitman et al., 1986). The rapid growth of *M. maripaludis* compared to *Methanosarcina* is advantageous for genetic experiments and recombinant protein expression. *M. maripaludis* S2 is the strain for which most reports of genetic manipulation utilize; however, *M. maripaludis* JJ has also been used successfully for this purpose. *Methanococcus maripaludis* can be grown on either H_2_/CO_2_ or formate, the latter of which provides an easier and safer alternative to dealing with high-pressure gases (Long et al., 2017). Genetic studies often utilize *M. maripaludis* strains lacking the gene for uracil phosphoribosyltransferase, which confers sensitivity to the base analog 6-azauracil and serves as a marker for negative selection (Costa et al., 2010; Sarmiento et al., 2011). Several shuttle vectors have been reported, and well-established methods exist for markerless mutagenesis and recombinant protein expression (Whitman et al., 1997; Sarmiento et al., 2011). *M. maripaludis* plasmids utilize puromycin or neomycin resistance for positive selection. Transformation methods for *M. maripaludis* S2 utilize polyethylene glycol-mediated transformation (Tumbula et al., 1994; Sarmiento et al., 2011). However, natural transformation facilitated by type IV-like pili has been reported for *M. maripaludis* JJ (Fonseca et al., 2020). Notably, a highly efficient CRISPR/Cas system has recently been developed for *M. maripaludis* JJ that utilizes a bacterial Cas12a along with the native homology directed repair machinery (Bao and Scheller, 2021). The capacity for natural transformation as well as the available CRISPR/Cas technology for genetic manipulation makes *M. maripaludis* JJ an especially attractive host for future metabolic engineering applications. Finally, natural transformation *via* type IV-like pili has also been demonstrated in *Methanoculleus thermophilus* (Fonseca et al., 2020). Using an established plasmid employed for generating *M. maripaludis* gene deletions, the authors generated a *M. thermophilus* deletion strain lacking genes for pili to demonstrate that pili are essential for natural transformation (Fonseca et al., 2020). This is the first report of genetic manipulation in a methanogen from the order *Methanomicrobiales*.

*Methanocaldococcus jannaschii* is a hyperthermophilic methanogen (Jones et al., 1983) that was the first archaeal organism to have its genome sequenced (Bult et al., 1996). *Methanocaldococcus jannaschii* has the fastest doubling time of any methanogen (26 min) and grows optimally at 85°C (Jones et al., 1983). Recently, the first genetic tools for *M. jannaschii* were reported (Susanti et al., 2019). *Methanocaldococcus jannaschii* is resistant to antibiotics commonly used with other archaea, such as previously mentioned puromycin or neomycin, as well as the base analogs used for counter selection in other methanogens (Susanti et al., 2019). However, it was found to be sensitive to mevinolin and simvastatin. These compounds are competitive inhibitors of 3-hydroxy-methylglutaryl (HMG)-CoA reductase and thus overexpression of HMG-CoA reductase can be used as a selection marker (Susanti et al., 2019). Based on this, a suicide vector was developed for generating in-frame gene deletions in *M. jannaschii,* where the gene of interest is replaced with *hmgA*. Further, a similar strategy was used to place a gene of interest under the control of a strong promoter and to add an affinity tag on the chromosome for subsequent purification of the overexpressed protein (Susanti et al., 2019). *M. jannaschii* is transformed using heat shock without the need for chemical treatments, such as PEG in *M. maripaludis* S2 or liposomes in *M. acetivorans* (Susanti et al., 2019). This system is not yet as advanced as for the methanogens described above since only linearized suicide vectors have been used, which does not allow the investigation of potentially essential genes and is not compatible with most strategies for markerless mutagenesis. Additionally, heterologous protein expression in *M. jannaschii* has not yet been reported.

*Methanothermobacter marburgensis* and *Methanothermobacter thermautotrophicus* ΔH are two highly similar *Methanobacteriales* methanogens that were used as model hydrogenotrophic methanogens during early biochemical investigation of methanogenesis and MCR (Thauer, 1998). *Methanothermobacter* species have also been successfully employed in bioreactors for efficient methane production processes (Thema et al., 2019; Pfeifer et al., 2021). Thus, developing genetic manipulation tools for these organisms has been a major area of interest. Very recently, the first reliable system for *M. thermautotrophicus* ΔH was reported (Fink et al., 2021). *Methanothermobacter thermautotrophicus* ΔH was found to be amenable to plating on solid medium with good efficiencies, and neomycin was shown to be effective for selection. This allowed for the construction of a shuttle vector containing a full array of cloning sites, selections markers for *E. coli* and *M. thermautotrophicus* ΔH, and the β-galactosidase-encoding gene *bgaB,* which can be used as a reporter (Fink et al., 2021). Transformation of *M. thermautotrophicus* ΔH is possible *via* interdomain conjugation with *E. coli* S17-1. As a proof-of-concept, this system was utilized to heterologously express formate dehydrogenase, thus enabling *M. thermautotrophicus* ΔH to grow with formate as a substrate (Fink et al., 2021). This genetic system could potentially be very useful for the future heterologous expression of MCRs since established methods for MCR activation in *M. marburgensis* (see section below) would likely also be effective for MCRs isolated from *M. thermautotrophicus* ΔH.

## Considerations for Promoters, Operon Organization, and PTMs for Recombinant MCRs

An important factor to consider when designing an expression construct for recombinant MCR expression is which promoter to use. Promoters are essential for transcription and translation as, like eukarya, archaea possess more complex machinery for translation compared to bacteria (Lyu and Whitman, 2017). Common promoters used for heterologous protein expression in *M. acetivorans* include the MCR promoter (P*mcrB*) and associated tetracycline-inducible forms (Buan et al., 2011), while in *M. maripaludis* the histone promoter A (P*hmvA*) is often used (Sarmiento et al., 2011). An inducible *nif* promoter has also been used for *M. maripaludis* (Lie and Leigh, 2002; Chaban et al., 2007) and, very recently, an inducible expression system based on phosphate limitation was developed (Akinyemi et al., 2021). Notably, a plasmid containing the P*pst* promoter, which becomes activated under phosphate-limiting conditions, was used to express a recombinant *M. maripaludis* MCR in *M. maripaludis*, which represented 6% of the total protein content in a cell-free extract (140% increase compared to when using P*hmva*; Akinyemi et al., 2021). Additionally, the *mmpX* gene, which encodes the radical SAM methylase responsible for the methyl-arginine modification in McrA was successfully expressed. This is a particularly interesting result, as the expression of this protein using the constitutive promoter P*hmva* results in very low protein yields since it is apparently toxic to the cells (Akinyemi et al., 2021). Besides using previously employed constitutive or inducible promoters for recombinant MCR expression, another possibility is to utilize the native promoter for the MCR of interest. This has been done previously for the heterologous expression of ANME-1 MCR in *M. acetivorans* (Soo et al., 2016; discussed more below). If this strategy is chosen, it would be important to consider whether the heterologous host transcription/translation machinery is able to recognize the essential promoter elements present in the foreign promoter.

When designing a plasmid construct for heterologous MCR expression, it is also important to consider operon organization. ANME MCR operons generally lack one or both accessory proteins (e.g., *mcrBGA*—ANME-1 or *mcrBDGA*—*Ca.* M. nitroreducens) in the operon, while methanogens will always contain at least one MCR operon with both accessory proteins within the operon (Figure 5). Although it is still unclear how accessory proteins affect MCR assembly and/or activation, generating constructs for heterologous expression with accessory proteins may be necessary, even in the case where they are not present in the MCR operon of interest, especially since the accessory proteins may be organism-specific. Additionally, considering the post-translational machinery present in the heterologous host of choice will be important, as well as whether those enzymes will effectively recognize and correctly modify the recombinant enzyme. Since not all PTMs are consistent across different MCRs, additional and/or replacement PTM genes may need to be incorporated, which may potentially be toxic to the host.

## Methods for Obtaining an Activated MCR

Assuming successful expression, assembly, and post-translational modification of a recombinant MCR, the next consideration is obtaining the active form of the enzyme. The three oxidation states of F_430_ are well-described and can be observed *via* electron paramagnetic resonance (EPR) spectroscopy and UV–Vis spectrophotometry. These include the active MCR_red1_ in Ni(I) form, the inactive MCR_silent_ in the Ni(II) form, and the “ready” MCR_ox1_ in the Ni(III) form (Goubeaud et al., 1997; Duin et al., 2011). Many isolation and purification procedures will yield MCR with F_430_ in an inactive Ni(II) state, thus representing a major limitation for the enzymatic investigation of various MCRs.

The first successful MCR activation procedure involved incubating *M. marburgensis* cells with 100% H_2_ prior to harvesting and including 10 mM CH_3_-S-CoM to stabilize the enzyme during purification (Rospert et al., 1991). This resulted in MCR almost entirely in MCR_red1_ state, which can be used for subsequent enzymatic assays. Subsequent work has shown that HS-CoM can be used instead of CH_3_-S-CoM to achieve activation (Duin et al., 2011). Other successful efforts to activate MCR from *M. marburgensis* involved the treatment of cells with 80% N_2_/20% CO_2_ prior to harvesting, which resulted in the MCR_ox1_ form of the enzyme (Goubeaud et al., 1997). Upon incubation of purified MCR_ox1_ with Ti(III) citrate, EPR spectra revealed the conversion from MCR_ox1_ to MCR_red1_, and specific activity of the enzyme raised from 2 U/mg protein to 100 U/mg protein. The advantage of isolating MCR in the MCR_ox1_ form is that this strategy minimizes oxidation of MCR_red1_ to the MCR_silent_ Ni(II) state, which cannot be reduced to the active form *in vitro* with chemical reductants. Another protocol for activation of MCR from *M. marburgensis* involved treatment of cells with CO, which was shown to activate MCR at a significantly faster rate than treatment with H_2_ (Zhou et al., 2013). CO activation resulted in MCR_red1_ within 1 h of incubation, while H_2_ treatment required overnight incubation. Although effective, these described MCR activation protocols have primarily only been used successfully for MCR isolated from *M. marburgensis.*

Toward developing strategies for activating MCR from other organisms, it was reasoned that MCR activation could be achieved by controlling the ligation state of nickel through the addition of different chemical agents (Becker and Ragsdale, 1998). Thus, sodium sulfide was added to *Methanosarcina thermophila* cells prior to harvesting, which successfully elicited the MCR_ox1_ state (Becker and Ragsdale, 1998). Using ^35^S-labeled sulfide, the authors demonstrated that the sulfide enters the cell, binds to the nickel site in F_430_, and remains bound during purification. The amount of MCR_ox1_ was correlated to the amount ^35^S-labeled sulfide. Thus, based on the available protocols described so far, isolation of MCR as MCR_ox1_ using sodium sulfide treatment will likely be the most widely applicable to different MCRs isolated from various organisms. This is because ligating nickel to control the oxidation state should be independent from the metabolic state of the cell, which would allow for the control of the MCR oxidation state regardless of which organism and/or which methanogenic substrate is used.

*In vivo* MCR activation remains a poorly understood process. Early work provided initial insights into the cellular components responsible for the reduction of CH_3_-S-CoM to methane (see (Thauer, 2019) for a comprehensive discussion of these experiments). In 2014, complete activation of MCR_ox1_ and 65% activation of MCR_silent_ was achieved in the presence of dithiothreitol, ATP, component A2, and component A3a (Prakash et al., 2014). An important discovery for this work was that the heterodisulfide product promotes the inactivation of MCR and thus it is essential to isolate the activation process from the methane formation reaction (Prakash et al., 2014). Further, the authors characterized component A3a as a 700 kDa complex that includes an assortment of redox proteins as well as McrC (Prakash et al., 2014). Any attempts to activate MCR with a smaller version of this complex were unsuccessful. Although much is still unclear about the structural basis of the complex and how it operates to activate MCR, this is a major advancement toward understanding how methanogens are able to supply low potential electrons to reduce F_430_ to the active Ni(I). Additionally, the presence of McrC in this complex finally linked a functional role to McrC, one of two accessory proteins within methanogenic MCR operons. It will be essential to fully elucidate the activation proteins and cofactors required as well as to obtain information about specificity in order to engineer an effective heterologous host for expression of active MCRs.

## Examples of MCR Recombinant Expression

The first reported example of the heterologous expression of a recombinant MCR in a methanogen was for the ANME-1 MCR from the Black Sea mat (the same ANME-1 MCR for which the crystal structure is solved), which was expressed in *M. acetivorans* to engineer the methanogen to perform reverse methanogenesis using Fe(III) as an electron acceptor (Soo et al., 2016). The authors found that the strain expressing ANME-1 MCR consumed almost two times more methane compared to *M. acetivorans* with an empty vector. A further engineered air-adapted strain of *M. acetivorans* (Jasso-Chávez et al., 2015) containing ANME-1 MCR was used to generate electricity from methane in a microbial fuel cell containing other engineered microbes (McAnulty et al., 2017). Despite the critical importance of these studies toward the goal of activating methane for a range of biotechnology applications, it is still unclear whether the recombinant ANME MCR was necessary to significantly facilitate methane oxidation or, on a more fundamental level, how much of the recombinant MCR was produced in a complete and active form, especially since the methanogen lacks the 17^2^-methylthio-F_430_ utilized by ANME-1 MCR. Notably, another investigation demonstrated that wild-type *M. acetivorans* is also capable of growth on methane using Fe(III) as an electron acceptor (Yan et al., 2018), indicating that the native methanogenic MCR is also capable of methane oxidation.

Another significant study described the heterologous expression of the MCR from *M. okinawensis* in *M. maripaludis* (Lyu et al., 2018b). The recombinant MCR was cloned into the traditional *M. maripaludis* protein expression plasmid under the control of P*hmvA* and with a his-tag on the C-terminus of McrA. This resulted in a highly expressed and uniformly assembled recombinant MCR as determined *via* SDS-PAGE and MALDI-MS. Additionally, expression of a MCR hybrid construct, consisting of *mcrBDCG* from *M. okinawensis* and *mcrA* from *M. maripaludis* resulted in a MCR consisting of the exact gene products from the hybrid construct without any components from the chromosomally encoded MCR. This supports the idea of an ordered MCR assembly, where MCR is simultaneously transcribed and translated (Lyu et al., 2018b). The PTMs were shown to be installed correctly for the heterologously produced *M. okinawensis* MCR and ~ 20% of the recombinant enzyme contained F_430_. Interestingly, the portion of the recombinant MCR that did not contain F_430_ was found to be associated with McrD, while the portion that did have F_430_ largely lacked McrD. This further supports a role for McrD in F_430_ delivery. Since only a fraction of the recombinant MCR contained F_430_, this suggests that potentially the F_430_ biosynthesis machinery cannot keep up with supplying F_430_ to the additional MCR being produced in the cell. Alternatively, the protein(s) potentially required to interact with McrD and/or MCR for F_430_ incorporation may not recognize the non-native McrD/MCR. If F_430_ biosynthesis was the bottleneck, one may expect that the native MCR would also have lower F_430_ incorporation. The authors found that the native MCR expression was not significantly affected in the presence of the recombinant MCR, but they did not report whether the F_430_ incorporation into native MCR was impaired. Finally, the purified heterologously produced MCR was found to exhibit low but detectable methane formation activity, where the authors point out that methods to activate *M. marburgensis* MCR *in vitro* do not appear to be effective for methanococcal MCR (Lyu et al., 2018b). It is important to note that *M okinawensis* and *M. maripaludis* are very closely related organisms, so it is unclear what the threshold of relatedness will be with respect to heterologous expression of MCRs from diverse organisms in model methanogens, such as *M. maripaludis* and *M. acetivorans*.

## Conclusion

Although the field has seen many significant advancements since the initial discovery of MCR, many questions remain that need to be addressed, especially toward the development of robust heterologous expression systems for diverse MCRs and ACRs. Specifically, the functions of unique PTMs and F_430_ modifications need to be elucidated, which will require *in vitro* kinetic studies with mutated MCRs in the presence vs. absence of modified F_430_s. Further, the activation of MCR remains a poorly understood process, including the roles of specific proteins identified in complex A3a (Prakash et al., 2014) as well as the ATP costs of this process. There is still very little known about the putative accessory proteins necessary for MCR assembly—McrD may serve as an F_430_ chaperone for delivery to the MCR active site, but no other proteins potentially involved in assembly have been discovered. In terms of MCR kinetics and mechanism, it is important to emphasize that the vast majority of what is known results from studies on a single MCR from *M. marburgensis*. Since the substrate specificity and catalytic efficiency of even closely related enzymes can vary, it will be important to develop tools to study the enzymatic capabilities of other MCRs and related ACRs.

## Author Contributions

AG and KA wrote and edited the manuscript. All authors contributed to the article and approved the submitted version.

## Funding

MCR research in the Allen lab is funded by the DOE Office of Science (DE-SC0022338).

## Conflict of Interest

The authors declare that the research was conducted in the absence of any commercial or financial relationships that could be construed as a potential conflict of interest.

## Publisher’s Note

All claims expressed in this article are solely those of the authors and do not necessarily represent those of their affiliated organizations, or those of the publisher, the editors and the reviewers. Any product that may be evaluated in this article, or claim that may be made by its manufacturer, is not guaranteed or endorsed by the publisher.
